# Effectiveness of Bedside Clinical Screening Tools in Predicting Short-Term Neurodevelopmental Delay Among Very-Low-Birth-Weight Pre-terms: A Prospective Observational Study

**DOI:** 10.7759/cureus.20355

**Published:** 2021-12-12

**Authors:** Jacquilyne Kharlukhi, Udayakumar Narasimhan, Saji James, Fatima Shirly Anitha, Sanmathi Suresh, Siri Ishwarya Polina

**Affiliations:** 1 Pediatrics, Sree Balaji Medical College and Hospital, Chennai, IND; 2 Pediatrics, Sri Ramachandra Institute of Higher Education and Research, Chennai, IND

**Keywords:** cdc grading, vlbw, screening tools, pre-term, neurodevelopmental delay, neonatal neurological examination

## Abstract

Background: Pre-term births are associated with increased risk of various morbidities, especially neurological. Early detection and early intervention to prevent these morbidities will have immediate and long-term benefits to the individuals and society at large. However, the screening and assessment tools, including both clinical and radiological, are not uniformly available in resource-poor settings. The present study was carried out to evaluate the validity of the clinical screening tools for detecting neurodevelopmental delay among very-low-birth-weight (VLBW) pre-term babies.

Methods: This prospective observational study was undertaken in the child development unit of a tertiary care hospital from July 2015 to October 2017. All pre-term VLBW neonates admitted in level III Neonatal Intensive Care within the first 24 hours of life were included in the study. They were subjected to Hammersmith Neonatal Neurological Examination (HNNE) and magnetic resonance imaging (MRI) of brain at term-equivalent age. Subsequently, the same group was followed up with Amiel-Tison (AT) angles, Child Development Centre (CDC) grading for sitting developed at Trivandrum, Kerala, India, and Denver Development Screening Test (DDST-II gross motor) at eight months corrected age, and their outcomes were analyzed.

Results: 17.9% of the ex-preterm were abnormal as per HNNE evaluation at term-corrected age. At short-term follow-up screening, 13.8% were found to be abnormal based on AT angles, while 35.2% were found to be abnormal as per CDC grading and 30.4% were found to have risk for delay as per DDST-II (gross motor). A high level of sensitivity (93.6%) and positive predictive value (91.2%) was observed for HNNE at term equivalent with MRI brain assessment considered as gold standard. Among the follow-up screening tools, CDC grading for sitting, AT angles, and DDST-II had high sensitivity (>85%).

Conclusion: The combination of HNNE along with radiological assessment at term-corrected age can be considered as appropriate for predicting long-term neurodevelopmental outcome in VLBW pre-term infants. During follow-up, simple tools like CDC grading for motor milestones, AT angles, and DDST-II may be utilized if facilities for standard assessment are not available as in resource-poor settings.

## Introduction

Babies born before 37 weeks of gestation are referred to as "pre-term”. Very-low-birth-weight (VLBW) babies are those who are born with a birth weight less than 1500 grams [1]. Several studies have consistently documented that pre-term neonates are at increased risk for mortality and various morbidities and developmental disabilities ranging from cerebral palsy, intellectual disability, blindness, learning disabilities, autism spectrum disorders, and attention-deficit disorders. According to global statistics, India tops the list of pre-term births, with a pre-term birth rate of 10%-14% as of 2014. In comparison with the international rates, the prevalence of pre-term births constitutes 24% of the total pre-term births worldwide [2].

Pre-term births are the leading cause of early neonatal deaths and the second most common cause of under-five deaths [3]. In the era of modern medicine, a wide range of technical and technological advancements help with effective treatment options, which decrease mortality rates [4,5]. Early identification and early intervention of pre-term babies with developmental delay have several immediate and long-term benefits in terms of neurodevelopmental outcomes to the individual and society. It is, therefore, essential to detect these neurological morbidities as early as possible to improve outcomes. High-risk follow-up of the pre-term infants provides an excellent opportunity for early identification and referral for neurodevelopmental delays with the use of standardized neurological examinations.

Neurological examinations suitable for young infants and toddlers have been developed for clinical care and research settings. These include assessment tools like the Hammersmith Short Neonatal Neurological Examination (HNNE), Prechtl’s General Movements Assessments, Hammersmith Infant Neurological Examination (HINE), Alberta Infant Motor Test, Bayley Scale of Infant Development-III (BSID III), and Development Assessment Scale for Indian Infants (DASII). The clinical screening tools include Amiel-Tison angles (AT), Child Development Centre (CDC) grading for motor milestones (developed by CDC, Trivandrum, Kerala), and Denver II Developmental Screening Test (DDST-II gross motor). These tools are well studied for identifying babies at risk for developmental delay; however, their availability in resource-poor settings is a matter of concern.

With this background, the present study was carried out to find out the neurological outcomes of pre-term babies weighing less than 1500 grams (VLBW pre-term) using HNNE at term-corrected age, and using AT angles, CDC grading for sitting, and DDST-II (gross motor) at eight months corrected age follow-up in a tertiary child development unit where structured early stimulation program is carried out as a routine procedure.

## Materials and methods

Study setting and selection of participants

This study was carried out as a prospective observational study in the child development unit of our tertiary care institution for a period of 28 months between July 2015 and October 2017. Neonates were enrolled for the study at the time of discharge from the neonatal intensive care unit (NICU). All pre-term infants weighing less than 1500 grams, admitted within the first 24 hours of life, were included. Babies admitted after 24 hours of life in the NICU were excluded as we wanted to exclude extramural pre-terms. A total of 170 pre-term infants were included in the study.

Ethical approval and informed consent

Approval was obtained from the Institutional Ethics Committee of Sri Ramachandra Institute of Higher Education and Research prior to the commencement of the study (IEC Ref no: CSP-MED/15/AUG/24/05). Each participant’s parents were explained in detail about the study and informed consent was obtained prior to the data collection.

Data collection

Sex, birth weight, length, head circumference, gestational age, Apgar score at 1 minute, and cranial ultrasound (USG) done at days 0, 7, and 28 days of life were all recorded in a structured proforma. Magnetic resonance imaging (MRI) brain was carried out at term-corrected age of 40 weeks among the study population. All parents were explained about the early mother-based stimulation program of CDC, Trivandrum. HNNE was performed on all the babies and their results were recorded [6]. This tool assessed the posture, tone, movement, reflexes, and motor milestones in the study participants. HNNE is a simple and scorable method for assessing neurological status in pre-term babies at term-corrected age. If any infant was found to be at risk for developmental delay, then the infant was put on early intervention program of CDC, Trivandrum. At eight-month follow-up, all the study participants underwent tests with clinical screening tools like AT angles to evaluate tone abnormalities, CDC grading for sitting and DDST-II to assess gross motor function, and the individual data of each infant were collected [7-9]. AT angle is a simple and effective tool to assess the tone and neurodevelopmental outcomes before the first year of age and thereby provide interventions. CDC motor grading is a simple tool developed by Nair et al., which can be used by any health worker with minimal training and is validated against the standard tools in infants. DDST-II is a simple screening tool that can be used with training and is less time consuming than the other standard assessment tests. AT angle has a sensitivity of 41.15%, specificity of 83.74%, and an accuracy of 70.2%; CDC grading for motor milestones has a sensitivity of 42.71%, specificity of 82.50%, and an accuracy of 70.53%; and DDST-II has a sensitivity of 30.93%, specificity of 79.02%, and an accuracy of 63.62% as compared to DASII [9]. All examinations were performed by the primary investigator after completing initial training at the facility.

Data analysis

Statistical analyses were performed to assess the validity of clinical assessment tools like HNNE at term-corrected age and other clinical screening tools (AT angles, CDC grading for sitting, and DDST-II [gross motor]) at eight months corrected age follow-up in screening for neurodevelopmental delay. Calculations were done using Graphpad Prism 5 (Graphpad software Inc., San Diego, CA, USA). Fisher’s exact test and chi-square test were used to analyze all categorical variables. A probability value (p) less than 0.05 was considered statistically significant.

## Results

At the start of the study 170 pre-term infants were enrolled and HNNE was performed at term-corrected age. During the follow-up period, 25 participants lost to follow-up and, therefore, follow-up data were recorded for only 145 participants. MRI brain at term-corrected age was performed in 96 participants. The mean gestational age of the study participants was 30.26 (±2.21) weeks, while the mean birth weight was 1216 (±240.3) grams. The mean length of the study participants was 38.55 (±3.34) cm, while the mean head circumference was 27.43 (±2.82) cm. Majority of the participants were males (59.3%) and the birth weight was between 1000 and 1500 grams (80%). Over 70.3% of the participants had a normal Apgar score at birth (Table 1).

**Table 1:** Background characteristics of the study participants.

S. No	Characteristics	Frequency (n=145)	Percentage
1	Gender		
	Male	86	59.3
	Female	59	40.6
2	Birth weight (in grams)		
	1000-1500	116	80
	750-1000	24	16.5
	<750	5	3.5
3	Weight for gestational age		
	Large for gestational age	3	2.1
	Appropriate for gestational age	113	77.9
	Small for gestational age	29	20
4	Apgar score at birth		
	>7	102	70.3
	3-7	41	28.3
	<3	2	1.4

At term equivalent, 17.9% of the VLBW pre-terms had abnormal examination as per HNNE examination. During follow-up at eight months corrected age, 13.8% were found to be abnormal based on AT angles, while 35.2% were found to be abnormal as per CDC grading for sitting and 30.4% were found to be at risk for delay as per DDST-II (gross motor) (Table 2).

**Table 2:** Follow-up evaluation of neurodevelopmental delay among the study participants. CDC, Child Development Centre;  DDST-II, Denver II Developmental Screening Test.

S. No	Tools	Frequency (n=145)	Percentage
1	Amiel-Tison angles		
	Normal	125	86.2
	Abnormal	20	13.8
2	CDC grading for sitting		
	Normal	94	64.8
	Abnormal	51	35.2
3	DDST-II (gross motor)		
	Normal	101	69.6
	At risk for delay	44	30.4

Radiologically, cranial USG at 28 days showed abnormalities in 14.5% of the participants while MRI brain was abnormal among 16.7% of the participants at term equivalent (Table 3).

**Table 3:** Radiological findings of neurodevelopmental delay. MRI, magnetic resonance imaging.

S. No	Radiological investigation	Frequency (n)	Percentage
1	Ultrasound		
	Total	145	
	Normal	124	86.5
	Abnormal	21	14.5
2	MRI		
	Total	96	
	Normal	80	83.3
	Abnormal	16	16.7

On comparing the parameters based on birth weight, the ex-preterm VLBW babies found to be abnormal as per HNNE examination belonged to the birth weight of <1000 grams (29.1%), while the same as per AT angles, CDC grading for sitting, and DDST-II (gross motor) evaluation at eight months corrected age follow-up belonged to the birth weight of <1000 grams (31%, 44.8%, and 41.4%, respectively) (Table 4).

**Table 4:** Correlation between birth weight and neurodevelopmental delay. HNNE, Hammersmith Short Neonatal Neurological Examination; CDC, Child Development Centre; DDST-II, Denver Developmental Screening Test II.

S. No	Tools	Birth weight 1000-1500 grams		Birth weight <1000 grams		Chi-square	p-Value
		Frequency (n)	Percentage	Frequency (n)	Percentage		
1	HNNE at term equivalent						
	Total	116		29		9.8	0.0050
	Normal	101	87.1	18	62.1		
	Abnormal	15	12.9	11	37.9		
2	Amiel-Tison angles at 8 months follow-up						
	Total	116		29		9.06	0.0056
	Normal	105	90.5	20	68.9		
	Abnormal	11	9.5	9	31.1		
3	CDC grading for sitting at 8 months follow-up						
	Total	116		29		1.48	0.278
	Normal	78	67.2	16	55.2		
	Abnormal	38	32.8	13	44.8		
4	DDST-II (gross motor) at 8 months follow-up						
	Total	116		29		2.08	0.177
	Normal	84	72.4	17	58.6		
	Abnormal	32	27.6	12	41.4		

The validity of various clinical screening tools was assessed in comparison with MRI brain done at term-corrected age, which was considered as the gold standard. A high level of sensitivity (93.6%), specificity (61.1%), and positive predictive value (91.2%) was observed for HNNE done at term-corrected age. Among the clinical and screening tools, AT angles, DDST-II (gross motor), and CDC grading for sitting had good sensitivity (>85%) but with low specificity ranging from 23.5% to 38.5%. Overall, sensitivity was the highest for HNNE at term equivalent (93.6%) (Table 5).

**Table 5:** Validity of clinical assessment tools in screening of neurodevelopmental delay. PPV, positive predictive value; NPV, negative predictive value; AT, Amiel-Tison angle; MRI, magnetic resonance imaging; HNNE, Hammersmith Short Neonatal Neurological Examination; CDC, Child Development Centre; DDST, Denver Developmental Screening Test.

S. No	Developmental tools				Validity represented as % (95% confidence interval)			
					Sensitivity	Specificity	PPV	NPV
1	MRI	HNNE			93.6 (93.4–93.8)	61.1 (60.4 –61.8)	91.2 (91.1–91.4)	68.7 (68–69.5)
		Normal	Abnormal	Total				
	Normal	73	7	80				
	Abnormal	5	11	16				
	Total	78	18	96				
2	MRI	Amiel-Tison angles at 8 months follow-up			86.7 (86.5–87)	38.5 (37.6–39.3)	90 (89.8–90.2)	31.3 (30.5–32)
		Normal	Abnormal	Total				
	Normal	72	8	80				
	Abnormal	11	5	16				
	Total	83	13	96				
3	MRI	CDC grading for sitting at 8 months follow-up			87.1 (86.8–87.4)	23.5 (23.1–24)	67.5 (67.2–67.8)	50 (49.2–50.8)
		Normal	Abnormal	Total				
	Normal	54	26	80				
	Abnormal	8	8	16				
	Total	62	34	96				
4	MRI	DDST at 8 months follow-up			86.6 (86.3–86.8)	24.1 (23.6–24.6)	72.5 (72.2–72.8)	43.7 (43–44.5)
		Normal	Abnormal	Total				
	Normal	58	22	80				
	Abnormal	9	7	16				
	Total	67	29	96				

## Discussion

Long-term neurodevelopmental delay is an established sequela of pre-term birth. In other words, the lower the gestational age and birth weight, the more is the risk for developmental delay [10-12]. Apart from neurodevelopmental delay, pre-term infants are more likely to have difficulties in attention, poor peer interaction, emotional and conduct problems, and autism spectrum disorders [13-16]. Visual impairment is also a long-term neurodevelopmental sequela in pre-term infants [16]. Therefore, early identification and follow-up of pre-term babies at risk result in better outcomes [17]. Hence, the need for early evaluation of these neonates and monitoring the progress in their early growing years is essential to start early intervention.

The mean gestational age of our VLBW pre-terms was 30.26 (±2.21) weeks and mean birth weight was 1216 (±240.3) grams. In a meta-analysis study done by Jorrit F. de Kieviet et al., the mean birth weight was 1060 (±207) grams and the mean gestational age was 28.2 (±1.5) weeks [18]. In another study done in New Jersey by Thomas H et al., the mean birth weight was 1393 (±406) grams and the mean gestational age was 31.3 (±4.1) weeks [19]. The findings were comparable to the present study. Birth weight significantly impacts the neurodevelopmental outcomes of pre-term babies and this has been emphasized in the meta-analysis by de Kieviet et al. [18]. In the present study, 77.9% were found to be appropriate for gestational age (AGA), 20% were small for gestational age (SGA), and 2.1% were large for gestational age (LGA). In a study done in VLBW babies in Missouri by Fernando A et al., 27.4% were term SGA, 61.3% were preterm AGA, and 11.3% were preterm SGA [20]. Majority of the participants in the present study (70.3%) had an Apgar score >7 at 1 minute and the findings were similar to the study done by Sykes et al. [21].

In the present study, 17.9% infants had an abnormal HNNE at term-corrected age. At eight months corrected age follow-up, 35.2% had abnormal CDC grading for sitting (p = 0.0013), and 30.4% were at risk for delay in DDST-II (gross motor) (p = 0.02) and significantly correlate with abnormal HNNE at term. In a study done by Amess P et al., it was found that, of the 14.6% with suboptimal score at term equivalent, 7.3% had optimal and 7.3% had suboptimal scores at 12-month follow-up based on Hammersmith Infant Neurological Examination (HINE) [22]. Abnormal HNNE at term-corrected age more significantly correlated with birth weight less than 1000 grams (p = 0.005). In this study, 13.7% were found to be abnormal at eight months corrected age by AT angles. There was a significant correlation between birth weight <1000 grams and 1000-1500 grams with abnormal AT angles at eight-month follow-up (p = 0.0001). In a study done by Sudha S et al., it was found that AT angles were more sensitive in detecting abnormal motor development at three, six, and nine months but lost its advantage at 12 months [23]. In a study done by Kanya Mukhopadhyay et al., 24% were abnormal according to AT angles, similar to the present study [24].

HNNE at term equivalent had high sensitivity (93.6%) and lower specificity (61.2%) when compared with term MRI brain which is considered as the gold standard. Among the tools administered at eight-month-corrected-age follow-up, AT angles, CDC grading for sitting, and DDST-II (gross motor) had good sensitivity (>85%) with lower specificity (23.5-38.5%). Serial USG cranium done during neonatal period can identify the pre-term babies at risk, is an inexpensive tool, easily done, and accessible even in remote neonatal units. In this study, 14.5% had abnormal USG findings (grades III and IV). MRI was feasible in only 96 of the study participants and it was found that MRI was abnormal in 16.6%. In a study done by Rademaker et al. [25] and Bayram et al. [26], 60.6% infants showed abnormal MRI findings. A study done by Setänen et al. [27] has documented that neurological examination at term-equivalent age improves the negative and positive predictive values of brain MRI for neurosensory impairment from 99% to 100%, and from 28% to 35%, respectively [27]. MRI brain is a more sensitive test and provides high-resolution non-invasive imaging of the brain, but is expensive and not generally available in rural areas. Therefore, these bedside clinical screening tools that have good sensitivity and are simple, valid, and cost-effective measures can be utilized in resource-poor settings to guide appropriate referral and to initiate early intervention programs.

There are certain limitations in the study including it being a single urban center study, all the preterm babies did not have MRI brain done at the term-corrected age, and also some babies were lost to follow-up. Since the CDC grading for sitting is a predominantly motor screening tool, we looked at motor domain only. Another limitation of the study is that it looked at the short-term outcome of eight months. Hence, large-scale multi-centric, long-term studies, including both urban and rural centers, are needed to further validate these findings.

## Conclusions

The present study has highlighted the effectiveness of various bedside clinical screening tools for identifying short-term neurodevelopmental delay in the context of resource-limited settings. Results showed higher sensitivity for HNNE at term-corrected age in identifying risk for neurodevelopmental delay and also better sensitivity for AT Angles, DDST-II (gross motor), and CDC grading at short-term follow-up. Hence, it may be practical to use HNNE at term-corrected age coupled with radiological examinations such as MRI or USG, depending on the feasibility and available resources for early detection of risk for neurodevelopmental delay. In the follow-up period, simple tools like AT angles, CDC grading for sitting, and DDST-II (gross motor) may be utilized if facilities for standard assessment are not available.
